# Silicone-Induced Granulomatous Reaction Causing Severe Hypercalcemia: Case Report and Literature Review

**DOI:** 10.1155/2019/9126172

**Published:** 2019-01-08

**Authors:** Gulshan Man Singh Dangol, Hilmer Negrete

**Affiliations:** ^1^Department of Internal Medicine, St. Elizabeth Health Center, Youngstown, Ohio, USA; ^2^Department of Internal Medicine, Northeastern Ohio Medical University, Rootstown, Ohio, USA

## Abstract

A 67-year-old woman presented to the hospital with complaints of abdominal pain. Physical exam was significant for signs of severe dehydration, mild epigastric tenderness and multiple non-tender hard nodules over her arms and thighs. Incidental finding of severe hypercalcemia led to negative workups for hyperparathyroidism, vitamin D intoxication, and malignancy. However, elevated levels of 1,25-hydroxy vitamin D raised the possibility of granulomatous diseases. Imaging and patient report revealed silicone-induced foreign body granulomatous reaction as the cause of hypercalcemia. Use of silicone for cosmetic enhancement of body contours can result in siliconomas, severe hypercalcemia, and complications. Treatment is unestablished for this condition. Increasing prevalence of cosmetic enhancement should prompt vigilance for this rare disease entity. Providers should counsel and educate individuals undergoing such procedures.

## 1. Introduction

Hypercalcemia is a common electrolyte abnormality with multiple etiologies, including hyperparathyroidism and malignancy [1]. Rare causes, accounting for less than 10% of cases, should be entertained when more prevalent etiologies have been eliminated. One such cause is extra-renal production of 1,25-hydroxy (OH) vitamin D (calcitriol) produced by silicone-induced foreign body granulomas, siliconomas [2–13].

Silicone, chemically inert, has been used for cosmetic enhancement either in the form of liquid silicone injections (LSI) or as silicone filled breast implants (SBI) [14]. In this article, we report a case of silicone-induced hypercalcemia, and review the literature, highlighting the pathophysiology and discuss therapeutic approaches for this exceedingly rare phenomenon.

## 2. Case

A 67-year-old woman presented to the hospital with complaint of burning epigastric pain over the past two months. Pain was intermittent, non-radiating, and associated with nausea and vomiting. She admitted to unintentional weight loss, approximately 30 pounds, during the past three months. She denied diarrhea, hematemesis, or melena. Significant past surgical history included bilateral silicone breast implants, exploratory laparotomy following gunshot wound with partial bowel resection, Billroth I gastrectomy following peptic ulcer disease, and partial thyroidectomy for a follicular adenoma.

**Figure 1:**
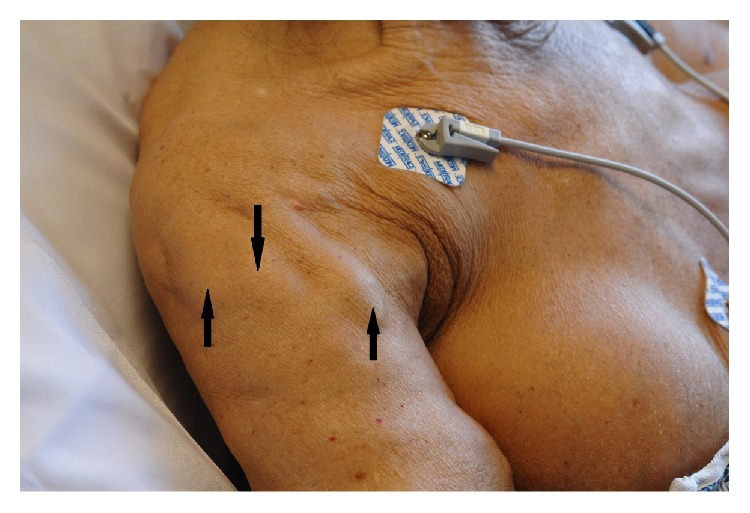
Palpable nodules (arrows) over the right arm.

**Figure 2:**
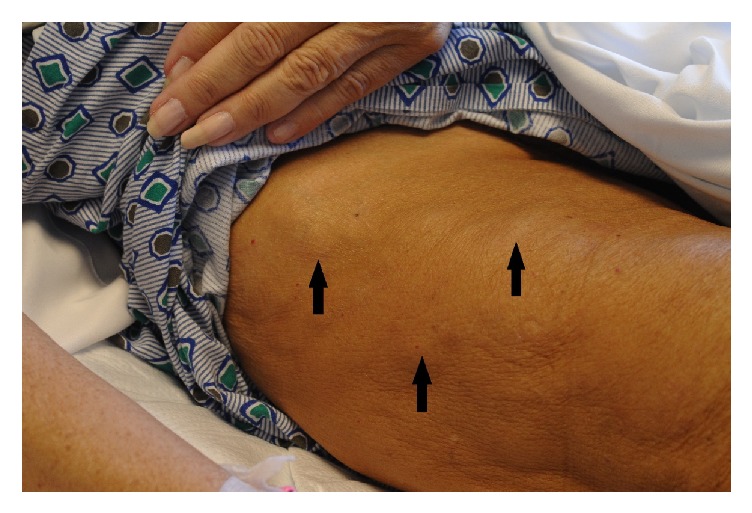
Palpable nodules (arrows) over the right thigh.

On examination, she appeared pale and severely dehydrated. Vital signs were 141 beats/minute, blood pressure 143/72 mm of Hg, temperature 97.4°F (36.3°C), and respiratory rate 18 breaths/minute. Current weight was 44 kg with a body mass index of 18.3 kg/m^2^. A right-sided thyroid lump was palpable, firm in consistency with regular margins, and moved with swallowing. Breast implants were “rock hard” in consistency with loss of distinct margin over the lower part of the right implant. Heart and lungs were clear to auscultation. Abdomen was soft with multiple surgical scars from previous surgeries. There was mild tenderness over the epigastric region without any guarding or rigidity. There was no rebound tenderness. Stool guaiac was weakly positive. Multiple fixed hard masses were palpable over both arms and thighs, but not painful, tender, or erythematous (Figures 1 and 2). On further questioning, she described right breast implant rupture diagnosed several years previous, but did not seek any medical advice because of financial issues.

**Figure 3:**
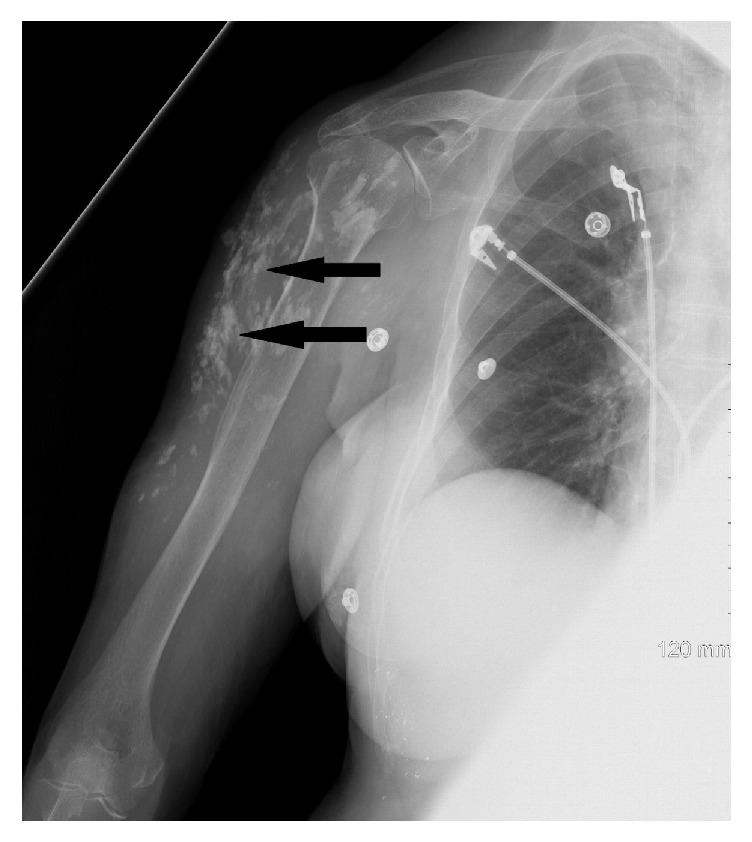
X-ray of the right arm showing soft tissue calcifications.

Admission blood work revealed severe hypercalcemia, Ca 18.4 mg/dL (normal: 8.6-10.2 mg/dl), and hyperphosphatemia, Phosphorus 6.8 mg/dL (normal: 2.5-4.5 mg/dl). Initial differential diagnosis for hypercalcemia included excessive antacid use, hyperparathyroidism, malignancy, and vitamin D intoxication. She denied any antacid use, but reported vitamin D 5000 U supplementation twice daily for the past five years. Parathyroid hormone was low, 13 pg/mL (normal 15-65 pg/ml); parathyroid hormone-related peptide,, was normal, <2 pmol/L (normal 0.0 – 3.4 pmol/L); serum and urine protein electrophoresis were both normal; and 25-hydroxy (OH) vitamin D was 40 ng/mL (normal 30-100 ng/mL). Esophagogastroduodenoscopy (EGD) and computed tomography (CT) of the chest and abdomen were negative for malignancy. EGD revealed moderate chronic gastritis; biopsies returned negative for* Helicobacter pylori* or intestinal metaplasia. Chest CT showed clear lung fields with stable bilateral pulmonary nodules, 3-5 mm, in the lower lobes. X-rays of the arms (Figure 3) and thighs revealed extensive soft tissue calcifications. Biopsy of the thyroid mass demonstrated benign-appearing follicular cells with abundant colloid, negative for malignancy. Abdominal CT was negative for any acute intra-abdominal pathology but showed universal calcinosis bilaterally affecting the musculature of the upper thighs. Colonoscopy demonstrated a hyperplastic polyp with evidence of melanosis coli; no malignant cells were seen.

**Figure 4:**
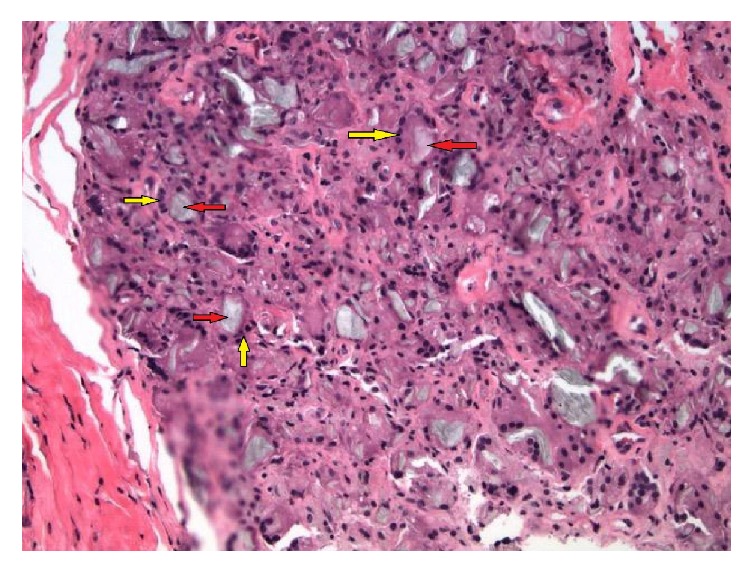
Microscopy of the biopsied mass from the left arm showing foreign body granulomatous reaction (high power view, 200 X). Foreign bodies (silicone materials) are shown by black arrows. These are surrounded by multi-nucleated giant cells (white arrows).

Serum 1,25-OH vitamin D was very elevated 290.7 pg/mL (normal 19.9-79.3 pg/mL), raising the suspicion of an underlying granulomatous disease. Angiotensinogen converting enzyme was mildly elevated, 137 U/L (Normal 9-67 U/L). Tissue biopsy of soft tissue calcifications was discussed; however, the patient refused. She was managed symptomatically and discharged home. Approximately three months later, the patient was re-admitted with hypercalcemia, 17.4 mg/dL. Abdominal CT scan revealed a 7 mm right sided ureteral calculus with mild hydronephrosis. Need for tissue biopsy was re-discussed with the patient and she agreed. Biopsy of the calcified mass on the left arm demonstrated dystrophic calcifications and foreign body granulomatous reaction (Figure 4).

During initial admission, the patient was managed with intravenous normal saline, subcutaneous calcitonin (4 units/kg) every 12 hours for three doses, and a dose of pamidronate (60 mg) intravenously. Despite treatment, the patient remained hypercalcemic, 12.8 mg/dL on hospital day 2. An additional dose of intravenous pamidronate (60 mg) was administered, and calcium level improved to 10.8 mg/dL. After hypercalcemia improved, the patient was discharged with consideration for low dose prednisone on an outpatient basis. However, she failed to follow-up.

On her second admission, three months later, she underwent right ureteral lithotripsy with placement of a ureteral stent, in addition to intravenous hydration, calcitonin, and bisphosphonates. After biopsy confirmed foreign body granulomatous reaction, plastic surgery was consulted for removal of breast implants and calcified granulomas. Given extensive extremity distribution of granulomas, surgical excisions were unfeasible. Removal of breast implants was considered but not done because of her financial constraints. The patient was started and maintained on prednisone 20 mg daily. Despite steroid initiation, the patient remained hypercalcemic and was readmitted after one month, a third time, with a serum calcium of 15 mg/dL.

**Figure 5:**
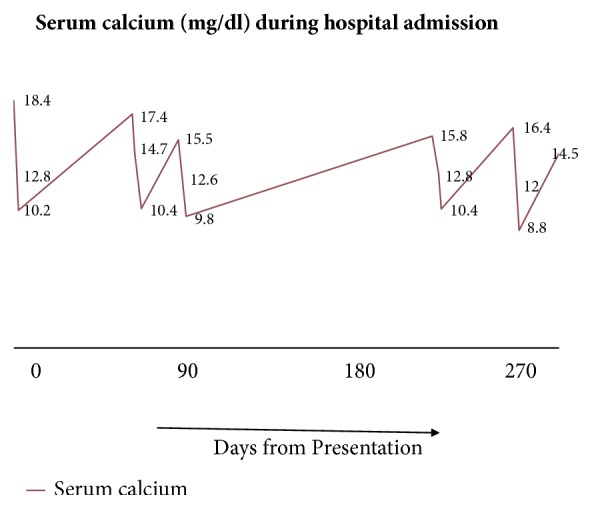
Graph showing serum calcium of patient during presentations.

For the past year, she has had multiple admissions with hypercalcemia and its complications (Figure 5). However, after steroid initiation, her hypercalcemic events were less severe.

## 3. Discussion

Silicone breast implants consist of a silicone gel enclosed within an outer shell, also known as an elastomer, which provides protection against contracture and rupture [15]. However, breast implant rupture may occur following trauma or during mammography. Once ruptured, silicone materials may disperse to distant sites, either through lymphatic or vascular systems.

The immune system can induce a foreign body reaction in response to administration of organic or inorganic materials, leading to chronic granulomatous inflammation. Epidemiological data has suggested higher rates of sarcoidosis in women receiving silicone breast implants [16]. Such reaction has a latent phase, usually following an acute inflammatory phase, and can last several years, prompting delayed clinical presentation. Typical presentation includes hardening of the skin and tissues, formation of lumps and nodules, and occasional ulcerations [17]. Silicone, either in the form of LSI or following SBI rupture, induces granulomatous reaction leading to the formation of foreign body tumors or siliconomas. Formation of siliconomas following silicone use was first described by Winer et al. in 1964 [18]. However, siliconomas causing hypercalcemia are extremely rare; only a handful of cases have been reported in the literature. Kozeny et al., in 1984, published the first incident of this rare condition [2]. Subsequently, thirteen cases have been reported (Table 1) [2–13]. Most of the cases reported were female; four were transgender. LSI were used in most cases with or without the implantation of SBI. In only one case, SBI was identified as the sole cause of hypercalcemia [10]. Also reported was the use of silicone with the help of transdermal ultrasound to remove wrinkles from the face and neck in one of the cases [5]. Hydration and steroids, with or without bisphosphonates, were the mainstay of treatment, but with improvement in only five cases [3, 5, 6, 12, 13]. Surgical excision of silicone granulomas helped normalize serum calcium in two of the reported cases, [9] and removal of breast implants resulted in improvement in one case [10]. The current case is the second case involving hypercalcemia solely secondary to SBI rupture. While our patient denied any LSI use, including on follow-up, silicone from implant rupture alone may be insufficient in quantity to cause the clinical and radiological findings. Typically, SBI ruptures contribute to local inflammation, and blood or lymphatic silicone transport is microscopic. However, distant multiple siliconomas in lower extremities following breast implant rupture have been described [19]. Among the cases reported, the current case has the highest serum calcium level reported. Use of steroid resulted in temporary improvement, and removal of granulomas and SBI were not pursued in our case.

The exact mechanism of silicone induced hypercalcemia is unknown. Formation of foreign-body granulomas by silicone may play a role in producing hypercalcemia. Granulomatous diseases, especially sarcoidosis, are known to cause calcitriol-related hypercalcemia. In these disorders, macrophages are known to produce 1-alpha-hydroxylase, responsible for the conversion of inactive vitamin D to its active form. Postulated pathophysiology includes the immunomodulatory effect of calcitriol, which decreases T-cell activity at the site of inflammation, probably by inhibiting interleukin-2 and *γ*-interferon [20]. In addition, immunohistochemical studies in vitro have demonstrated 1-alpha-hydroxylase activity in macrophages of sarcoid and foreign-body granulomas [20, 21].

Normally, the enzyme 1-alpha-hydroxylase is present in renal tubular cells, regulating the production of calcitriol. High serum levels of calcitriol cause negative feedback, leading to inhibition of 1-alpha-hydroxylase enzyme. However, these regulatory mechanisms are deficient in macrophages, resulting in unregulated 1,25-OH vitamin D production [18]. This unregulated extra-renal production of calcitriol causes increased intestinal absorption and calcium mobilization from bones, resulting in hypercalcemia.

Definitive treatment of silicone-induced hypercalcemia is unknown. The benefit of targeted monoclonal antibody treatment for molecular pathways, especially in interleukin production, is unknown, but may be beneficial in the fibrotic phase of silicone induced reactions [22]. Unlike sarcoidosis, where corticosteroids markedly improve production of 1,25-OH vitamin D, treatment with steroids is of limited value in silicone-induced hypercalcemia. While the addition of steroids and removal of foreign body granulomas and implants were helpful in some cases, no treatment modalities or response was noted in other cases. In our patient, steroids were of minimal benefit; removal of granulomas was unfeasible, given extensive dissemination. While steroids may decrease hypercalcemia severity, the adverse profile of steroids over long periods raises the question of possible risk versus benefit. Silicone-induced foreign-body granulomatous reaction should be considered in patients who have undergone cosmetic enhancement and developed hypercalcemia. Treating physicians should be aware of limited treatment modalities available for silicone hypercalcemia and educate individuals of the risk of silicone cosmesis.

## Figures and Tables

**Table 1:** Literature reported hypercalcemia cases following use of cosmetic silicone.

**Authors** **(year)**	**Age** ** (years)**	**sex**	**Site of silicone** **administration**	**Serum Calcium** **(mg/dL)** **∗** **∗**	**1,25-OH vitaminD** **(pg/mL)** **∗** **∗** **∗**	**Treatment** **received**	**Overall** **outcome**
Kozeny, et al. (1984)	33	Transgender	Silicone injections to face, breasts and both hips	14.7	85.0	Hydration and steroids	Serum calcium improved while on steroid, but increased after it was stopped

Singapuri, et al. (2010)	49	Female	silicone injections to gluteal region	14.6	NA*∗*	Hydration, bisphosphonates and steroids	Serum calcium stabilized

Ogbuagu, et al. (2010)	38	Female	Silicone breast implants; silicone injections to lips, hip and breasts	14.0	51.0	NA*∗*	NA*∗*

Ogbuagu, et al. (2010)	48	Transgender	Silicone injections to hips, face and breasts	11.8	63.0	NA*∗*	NA*∗*

Schanz, et al. (2012)	72	Female	Topical silicone applied over face and neck with transdermal ultrasound for wrinkles removal	12.1	39.0	Steroids	Serum calcium normalized

Agrawal, et al. (2013)	45	Transgender	Bilateral breast implants and silicone injections to buttocks	13.1	147.0	Hydration and steroids	Serum calcium normalized

Camuzard, et al. (2014)	63	Female	Silicone injections to buttocks and hips	13.0	65.0	Hydration, bisphosphonates and steroids	No improvement in serum calcium, patient refused surgical treatment

Visnyei, et al. (2014)	40	Transgender	Silicone injections to thigh and gluteal region	12.3	47.0	Hydration, bisphosphonates and steroids	Initial improvement in serum calcium, but noncompliance resulted in elevation of serum calcium

Edwards, et al (2015)	50	Female	Silicone injections to gluteal region	17.3	71.0	Steroid and pentoxiphylline; surgical removal of granulomas	Steroid and pentoxiphylline did not help; surgical removal of granulomas normalized serum calcium

Edwards, et al. (2015)	NA	Female	Silicone injections to gluteal region	11.5	89.0	Steroids and surgical excision of silicone granulomas	Surgical excision improved serum calcium levels

Melnick et al. (2016)	41	Female	Silicone injections to buttocks	13.5	84.0	IV hydration, pamidronate	Serum calcium improved at one year followup

Rodriguez, et al. (2017)	74	Female	Silicone breast implants	14.0	NA*∗*	Hydration, calcitonin and removal of breast implants	Improvement in serum calcium

Noreña, et al. (2017)	40	Female	Silicone injections to gluteal region	13.0	69.1	Hydration, calcitonin, bisphosphonates, sevelamer, chloroquine, and steroids	No improvement in serum calcium, patient died from complications of hypercalcemia

Hamadeh, et al. (2018)	35	Male	Silicone injections to shoulder, arms and forearms	13.1	19.3	Mass excision; hydration, and tapering course of steroids (40 mg daily for three weeks, tapered by 5 mg weekly thereafter)	Improvement in serum calcium

Dangol, et al. (current case) (2018)	67	Female	Silicone breast implants	18.4	290.7	Hydration, calcitonin, bisphosphonate and steroids	No improvement in serum calcium; multiple admissions with hypercalcemia and its complications

*∗*NA = not available.

*∗∗*Across the series, normal range for serum calcium was 8.2 – 10.3mg/dl.

*∗∗∗*Across the series, normal range for 1, 25-OH vitamin D was 15.0 – 86.5 pg/ml.
